# Cause-Specific Cardiovascular Risk Associated with Nonsteroidal Anti-Inflammatory Drugs among Myocardial Infarction Patients - A Nationwide Study

**DOI:** 10.1371/journal.pone.0054309

**Published:** 2013-01-30

**Authors:** Anne-Marie Schjerning Olsen, Emil L. Fosbøl, Jesper Lindhardsen, Charlotte Andersson, Fredrik Folke, Mia B. Nielsen, Lars Køber, Peter R. Hansen, Christian Torp-Pedersen, Gunnar H. Gislason

**Affiliations:** 1 Department of Cardiology, Copenhagen University Hospital, Hellerup, Denmark; 2 Department of Cardiology, the Heart Centre, Copenhagen University Hospital Rigshospitalet, Copenhagen, Denmark; 3 Duke Clinical Research Institute, Duke University Medical Center, Durham, North Carolina, United States of America; Marienhospital Herne - University of Bochum, Germany

## Abstract

**Background:**

Non steroidal anti-inflammatory drugs (NSAIDs) increase mortality and morbidity after myocardial infarction (MI). We examined cause-specific mortality and morbidity associated with NSAIDs in a nationwide cohort of MI patients.

**Methods and Results:**

By individual-level linkage of nationwide registries of hospitalization and drug dispensing from pharmacies in Denmark, patients aged >30 years admitted with first-time MI during 1997–2009 and their subsequent NSAID use were identified. The risk of three cardiovascular specific endpoints: cardiovascular death, the composite of coronary death and nonfatal MI, and the composite of fatal and nonfatal stroke, associated with NSAID use was analyzed by Cox proportional hazard analyses. Of 97,698 patients included 44.0% received NSAIDs during follow-up. Overall use of NSAIDs was associated with an increased risk of cardiovascular death (hazard ratio [HR] 1.42, 95% confidence interval [CI] 1.36–1.49). In particular use of the nonselective NSAID diclofenac and the selective cyclooxygenase-2 inhibitor rofecoxib was associated with increased risk of cardiovascular death (HR 1.96 [1.79–2.15] and HR1.66 [1.44–1.91], respectively) with a dose dependent increase in risk. Use of ibuprofen was associated with increased risk of cardiovascular death (HR 1.34[1.26–1.44]), whereas naproxen was associated with the lowest risk of (e.g., HR 1.27[1.01–1.59].

**Conclusion:**

Use of individual NSAIDs is associated with different cause-specific cardiovascular risk and in particular rofecoxib and diclofenac were associated with increased cardiovascular morbidity and mortality. These results support caution with use of all NSAIDs in patients with prior MI.

## Introduction

Non-steroidal anti-inflammatory drugs (NSAIDs) have been associated with increased cardiovascular risk and previously we have reported an increased risk of all-cause death and myocardial infarction (MI) with use of some NSAIDs among patients with prior MI [1], [2], [3]. As NSAIDs still are widely used in the general population [4] the cardiovascular risk associated with these agents seems to be a major public health issue, not least as even commonly used NSAIDs such as diclofenac and ibuprofen are associated with increased risk. In some countries these drugs are available as over-the-counter (OTC) drugs, and despite warnings related to unfavorable cardiovascular safety NSAIDs surveys have demonstrated increased sale of painkilling OCT medications in Denmark [5]. Because of the wide availability and use of NSAIDs, awareness of their proper use, dose, and potential side effects is warranted among health care providers as well as in the general population. Data on the cause-specific mortality associated with individual NSAIDs in patients with established cardiovascular disease are sparse. Investigation on specific cardiovascular causes of mortality and morbidity associated with NSAIDs in the highly selected population of prior MI patients can establish further details to the perception of the cardiovascular risk of these agents. Therefore the objective of this study was to clarify the cause-specific cardiovascular mortality and morbidity associated with the use of individual NSAIDs in a cohort of patients with prior MI.

## Methods

### Study design

The study was a nationwide registerbased cohort study in patients with prior MI in Denmark in the period 1997–2009.

### Data Sources

In Denmark each resident has a unique and permanent person identification number, which enables individual-level-linkage between nationwide registries.

The Danish National Patient Registry keeps records of all hospital admissions in Denmark since 1978 [6]. Each hospital admission is registered with one main discharge coding diagnosis, and if appropriate one or more supplementary diagnoses, according to the International Classification of Diseases (ICD) codes, until 1994 the 8^th^ revision (ICD-8) and from 1994 the 10^th^ revision (ICD-10).Vital status (dead or alive) was obtained from The Central Person Registry, which keeps records on vital status and registers all deaths within 14 days. From the National Causes of Death Register, in which immediate and underlying causes are recorded using the (ICD-10), the cause of death was procured.

Information on concomitant medication was obtained from The Danish Registry of Medicinal Product Statistics (national prescription registry), which keeps records on all dispensed drug prescriptions from Danish pharmacies since 1995. Each drug dispensing is registered according to an international classification of drugs, the Anatomical Therapeutical Chemical (ATC) system, as well as the date of dispensing, quantity dispensed, strength, formulation, and the affiliation of the physician issuing the prescription. Due to partial reimbursement of drug expenses by the Danish health care authorities, all pharmacies in Denmark are required to register each drug dispensing ensuring complete registration.

The data of socioeconomic status was available from Integrated Database for Labour Market Research. This database is based on information from taxed income gathered by government tax authorities and is therefore very accurate. Socioeconomic status was defined as the individual average annual income 5 years before the year of the index MI. For adjustment in the analyses, the population was divided in quintiles according to the annual income of patients.

Comorbidity was defined by using the Ontario acute myocardial infarction mortality prediction rule, modified for ICD-10 [7]. To further enhance adjustments for comorbidity, all discharge diagnoses were identified up to one year before the index MI hospitalization [8].

### Dose and Duration of Treatment

The national prescription registry does not include information on prescribed daily dosage of the medication. For each of the NSAIDs we created an algorithm in which a minimum, maximum and typical daily dosage of used medication was defined. For patients who had not been in treatment in the period proceeding the day of a prescription claim, the typical daily dosage was assigned and treatment length was calculated by dividing the amount of claimed medications by that daily dosage. For patients who were covered by a previous prescription claim at the time of claiming a new prescription, the daily dosage was reset and a new daily dosage was calculated as the amount of claimed medications during the preceding period divided by time between prescription claims. If calculated dosages exceeded the predefined highest daily dosages, patients were assigned the maximally dosages and exceeding tablets were assumed to be stored and consumed during the immediate period after duration of last prescription. The method used to determine the dose and treatment duration has been described previously [1], [9]. To analyse whether there was a dose-related response in risk of the three outcomes, the 2 COX-2 inhibitors (rofecoxib and celecoxib) and the 3 nonselective NSAIDs (ibuprofen, diclofenac and naproxen) were divided into low or high dosages. High dose was defined as being above the upper limit of the recommended minimal dose for each drug: ibuprofen, >1200 mg; diclofenac, 100 mg naproxen, >500 mg; rofecoxib, >25 mg; and celecoxib, >200 mg.

### Study Patients and follow-up

We identified a population of all patients with first-time admission for MI (ICD-10 I21-I22) from 1997 to 2009. First admission for MI implied that the National Patient Registry had not registered any prior admission for MI in the previous 19 years. Since the registry contains information on all hospital admissions from 1978, 19 years was the longest time we could track previous data on hospital admissions for patients admitted in 1997, and this length of history of prior hospitalizations was therefore used for all patients in our cohort [3]. To avoid selection bias in the exposure allocation due to the high mortality in relation to the MI, the cohort was restricted to individuals alive 30 days post discharge. Patients were followed until first occurrence of any of the following events: cardiovascular outcomes of interest (cardiovascular death, coronary death or MI, fatal or nonfatal stroke), death from other causes, emigration, or end of study period (December 31, 2009).

Furthermore we identified all claimed prescriptions of NSAIDs (ATC M01A) from the national prescription registry in the period after 30 days post-discharge from index MI. The selective cyclooxygenase (COX)-2 inhibitors, rofecoxib and celecoxib, and the most commonly used non-selective NSAIDs in Denmark, ibuprofen, diclofenac, and naproxen, were analyzed separately.

Concomitant use of beta-blockers (ATC C07), angiotensin-converting enzyme (ACE) inhibitors/angiotensin-2 receptor blockers ([ARBs] ATC C09), statins (ATC C10A), loop diuretics (ATC C03C), spironolactone (ATC C03D) and anti-diabetic drugs (ATC A10, a proxy for diabetes) [10] were also identified in the national prescription registry.

### Outcomes

The following outcome measures were used: Cardiovascular death (ICD 10 codes I00–I99) and two composite end points including coronary death and nonfatal MI (I20–I25 and I46), and fatal and nonfatal stroke (I60–I64), respectively. The diagnoses have been validated and found reliable with a sensitivity of 91% and a positive predictive value of 93% for the MI diagnosis [11] and the diagnoses of stroke (fatal and nonfatal), had a positive predictive value of 74% to 97% [12], [13].

### Statistics

The risk of cause-specific death associated with exposure to NSAIDs was estimated by incidence rates and time-dependent Cox proportional-hazard models. Exposure to NSAIDs was included as time-dependent covariates in the models, i.e., patients were only considered at risk, when they were exposed to the drug. Each individual could have multiple independent treatment courses with the same drug but also with different drugs. All models were adjusted for age, sex, year of index hospitalization, concomitant medication, comorbidity, and socioeconomic status. The proportional-hazard assumption, linearity of continuous variables, and lack of interaction were found to be valid unless otherwise indicated.

Cox proportional-hazard analyzes with time-dependent variables were performed using the Stata statistical package, version 11.0 (Stata Corp LP, College Station, TX). All other statistical analyzes and data management were performed with the SAS statistical software package, ver. 9.2 (SAS Institute Inc., Cary, NC).

### Ethics

The Danish Data Protection Agency approved this study (No 2007-41-1667). All individual level data were made available to us in an anonymized format so that specific individuals could not be identified. In Denmark, retrospective register studies do not require approval from the ethics committees.

## Results

From 1997 to 2009, a total of 128,418 patients were admitted with first-time MI; of these 97,698 (76.1%) were alive and had not experienced a study event 30 days after discharge and were therefore included in the study. Men comprised 64% of the study cohort and their mean age was 69 (standard deviation [SD] 13.0) years. Of the 97,698 patients included in the study 43,134(44.0%) filled at least one prescription of NSAID treatment during follow-up. Patients taking non-selective NSAIDs were younger and more often men compared with patients taking the selective COX-2 inhibitors rofecoxib and celecoxib. Patients not taking NSAID had more comorbidity. Otherwise no major differences between the treatments groups were found. Patients not using NSAIDs had similar age as those taking selective COX-2 inhibitors. A total of 3.7% of the patients received rofecoxib, 3.8% celecoxib, 26.8% ibuprofen, and 14.8% diclofenac. A detailed description of baseline characteristics of the study cohort and distribution between NSAID exposure groups is shown in Table 1.

**Table 1:** Baseline characteristics of the total study population and individual treatment groups.

				Exposure group*					
	Total population	No NSAID	Overall NSAID	Rofecoxib	Celecoxib	Ibuprofen	Diclofenac	Naproxen	Other NSAIDs
Characteristic	N (%)	N (%)	N (%)	N (%)	N (%)	N (%)	N (%)	N (%)	N (%)
Total patients	97,698(100.0)	54,564(55.8)	43,134(44.2)	3,596(3.7)	3,710(3.8)	26,158(26.8)	14,416(14.8)	2,382(2.4)	13,134(13.4)
Mean age (SD), y	68.9(13.2)	70.1(13.0)	66.1(12.9)	70.1(12.2)	70.1(11.9)	64.3(12.9)	64.6(12.5)	64.6(12.4)	67.4( 12.5)
Women	35,447(36.3)	20,853(37.5)	15,049(34.9)	1,746(48.6)	1,816(49.0)	8,373(32.0)	4,639(32.2)	722(30.3)	5,170(39.4)
Men	62,251(63.7)	34,726(62.5)	28,085(65.1)	1,850(51.5)	1,894(51.0)	17,785(68.0)	9,777(67.8)	1,660(69.7)	7,964(60.6)
Co-morbidity									
Cardiac arrhythmias	10,233(10.5)	6,761(12.4)	3,472(8.1)	346(9.6)	334(9.0)	1,839(7.0)	1,078(7.5)	153(6.4)	1,073(8.2)
Peripheral vascular disease	1,496(1.5)	975(1.8)	522(1.2)	54(1.5)	58(1.6)	279(1.1)	160(1.1)	23(1.0)	169(1.3)
Cerebral vascular disease	3,866(4.0)	2,596(4.8)	1,270(2.9)	145(4.0)	133(3.6)	660(2.5)	389(2.7)	51(2.1)	385(2.9)
Diabetes with complications	4,360(4.5)	2,696(5.0)	1,664(3.9)	146(4.1)	143(3.9)	994(3.8)	543(3.8)	91(3.8)	470(3.6)
Acute renal failure	830(0.9)	632(1.2)	198(0.5)	22(0.6)	17(0.5)	103(0.4)	47(0.3)	9(0.4)	46(0.4)
Chronic renal failure	1,319(1.4)	1,026(1.9)	293(0.7)	26(0.7)	20(0.5)	156(0.6)	75(0.5)	10(0.4)	71(0.6)
Malignancy	670(0.7)	476(0.9)	194(0.5)	10(0.3)	15(0.4)	105(0.4)	63(0.4)	6(0.3)	51(0.4)
Shock	967(1.0)	668(1.2)	299(0.7)	31(0.9)	21(0.6)	147(0.6)	92(0.6)	27(1.1)	89(0.7)
COPD	963(1.0)	634(1.2)	329(0.8)	30(0.8)	28(0.8)	163(0.6)	97(0.7)	19(0.8)	117(0.9)
Gastric ulcer	1,480(1.5)	958(1.8)	522(1.2)	81(2.3)	64(1.7)	256(1.0)	136(0.9)	20(0.8)	174(1.3)
Concomitant medical treatment									
Beta-blockers	72,416(74.1)	39,668(72.7)	32,748(75,9)	2,455(68.3)	2,568(69.2)	20,217(77.3)	11,159(77.4)	1,808(75.9)	9,839(74.9)
ACE inhibitors	44,171(45.2)	25,794(47.3)	18,377(42.6)	1,416(39.4)	1,510(40.7)	10,996(42.0)	5,937(41.2)	995(41.8)	5,494(41.8)
Statins	60,496(61.9)	33,942(62.2)	26,554(61.6)	1,469(40.9)	1,581(42.6)	16,951(64.8)	8,990(62.4)	1,434(60.2)	7,508(57.2)
ASA	56,780(58.1)	33,546(61.5)	23,234(53.9)	1,369(38.1)	1,472(39.7)	14,444(55.2)	7,537(52.3)	1,240(52.1)	6,614(50.4)
Clopidogrel	44,171(45.2)	26,877(49.3)	17,294(40.1)	597(16.6)	766(20.7)	11,015(42.1)	5,470(37.9)	865(36.3)	4,645(35.4)
Spironolactone	8,289(8.5)	5,328(9.8)	2,961(6.9)	285(7.9)	317(8.5)	1,637(6.3)	864(6.0)	161(6.8)	881(6.7)
Loop-diuretics	37,832(38.7)	23,125(42.4)	14,707(34.1)	1,629(45.3)	1,655(44.6)	8,084(30.9)	4,449(30.9)	787(33.0)	4,797(36.5)
Glucose lowering drugs	11,924(12.2)	7,057(12.9)	4,867(11.3)	424(11.8)	438(11.8)	2,913(11.1)	1,629(11.3)	256(10.7)	1,500(11.4)
PCI	31,811(32,6)	18,561(34.0)	13,250(30.7)	551(15.3)	600(16.2)	8,572(32.8)	4,401(30.5)	729(30.6)	3,563(27.1)
Socioeconomic factors									
Yearly family income in quintiles									
0	19,024(19.5)	11.028(20.2)	7,996(18.5)	1,040(28.9)	1,009(27.2)	4,376(16.7)	2.451(17.0)	457(19.2)	2,868(21.8)
1	18,939(19.4)	11,115(20.4)	7,824(18.1)	872(24.3)	929(25.0)	4,232(16.2)	2,442(17.0)	395(16.6)	2,622(20.0)
2	19,452(19.9)	11,042(20.2)	8,410(19.5)	635(17.7)	644(17.4)	5,108(19.5)	2,797(19.4)	429(18.0)	2,609(19.9)
3	19,900(20.4)	10,673(19,6)	9,227(21.4)	611(17.0)	658(17.7)	5,973(22.8)	3,271(22.7)	582(24.5)	2,642(20.1)
4 (highest)	20,383(20.9)	10,706(19.6)	9,677(22.4)	438(12.2)	470(12.7)	6,469(24.7)	3,455(24.0)	518(21.8)	2,393(18.2)

SD: standard deviation; MI: acute myocardial infarction; COPD: chronic obstructive pulmonary disease; ACE-inhibitors: angiotensin converting enzyme-inhibitors; ASA: acetylsalicylic acid; PCI: percutaneous coronary intervention.

* Each individual could have multiple treatment courses with the same NSAID and with different NSAIDs.

### Outcomes

During follow-up 23,321 patients died of cardiovascular causes, 26,513 died of coronary death or had a nonfatal MI, whereas 7,381 had a fatal or non-fatal stroke. Distribution of the specific causes of deaths according to exposure to NSAIDs is shown in Table 2.

**Table 2:** Distribution of Specific Primary Causes of Death in Post MI patients associated with exposure to nonsteroidal anti-inflammatory drugs (NSAID).

Specific Causes of Death	All Deaths (Excluding individuals who died during NSAID Exposure), n (%)	Deaths during NSAID Exposure, n (%)
All Causes	31,816(100%)	4,072(100%)
Malignancy (C00–C97)	5,867(18.4%)	1,129(27.7%)
Cardiovascular death (I00–I99)	21,069(66.2%)	2,252(55.3%)
MI (I21–I22)	3,480	592
Coronary death (I20 I25+I46)	2,612	1,460
Stroke (I61–I64)	3,899	173
Deaths from other causes	4,880 (15.3%)	691(17.0%)

ICD-10, International Classification of Diseases.

**Figure 1:**
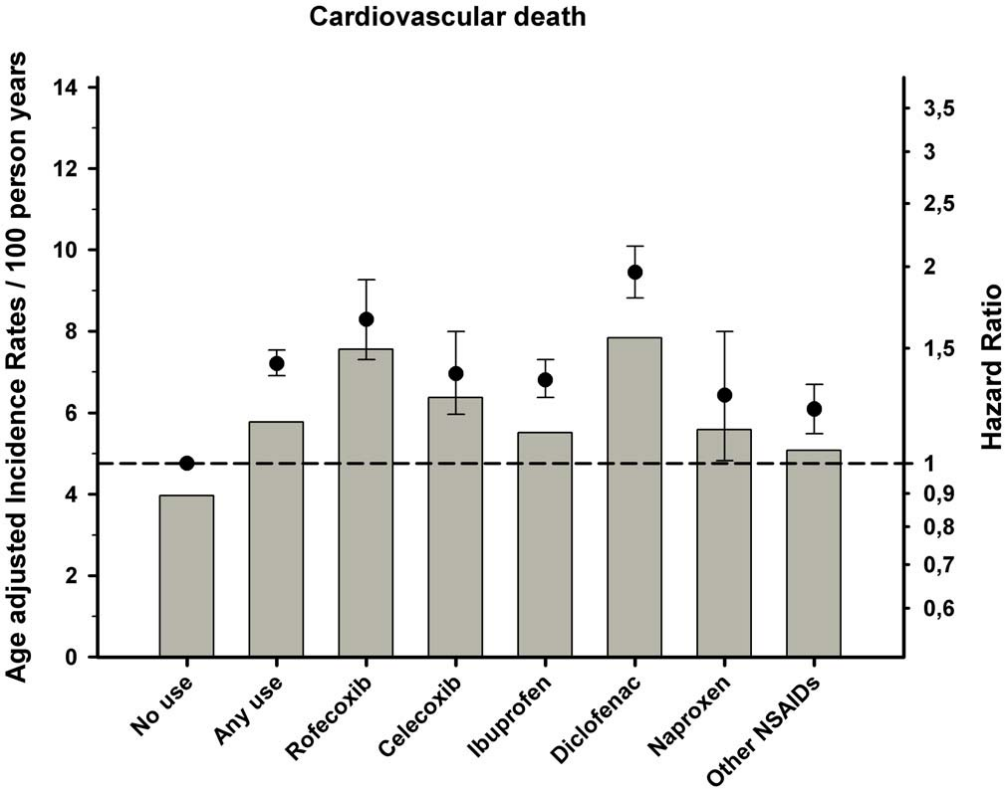
Cox proportional-hazard analysis and age adjusted incidence rates of cardiovascular death associated with use of NSAID treatment in patients with prior myocardial infarction (MI). The Cox proportional hazard model adjusted for, age, sex, year of MI, concomitant medical treatment, socioeconomic status, and comorbidity. Reference group: no use of COX-2 inhibitors or NSAIDs. Error bars indicate 95% CIs.

**Figure 2:**
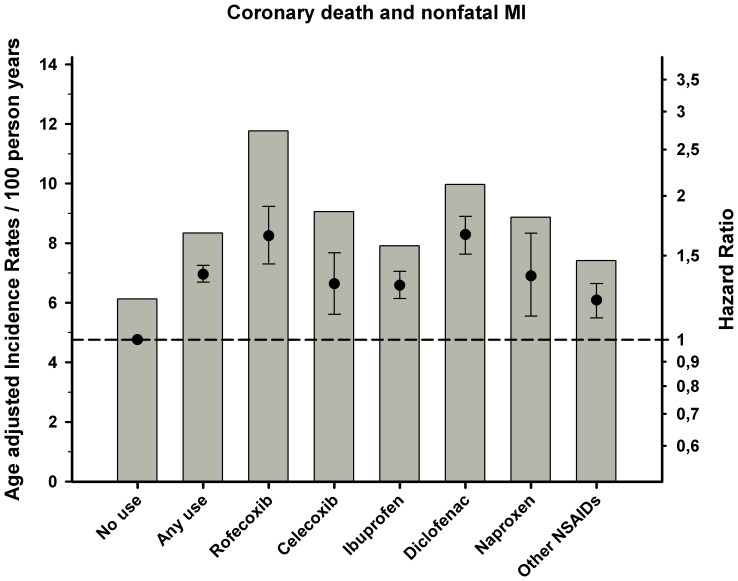
Cox proportional-hazard analysis and age adjusted incidence rates of coronary death and recurrent myocardial infarction (Re-MI) associated with use of NSAID treatment in patients with prior myocardial infarction (MI). The Cox proportional hazard model adjusted for, age, sex, year of MI, concomitant medical treatment, socioeconomic status, and comorbidity. Reference group: no use of COX-2 inhibitors or NSAIDs. Error bars indicate 95% CIs.

**Figure 3:**
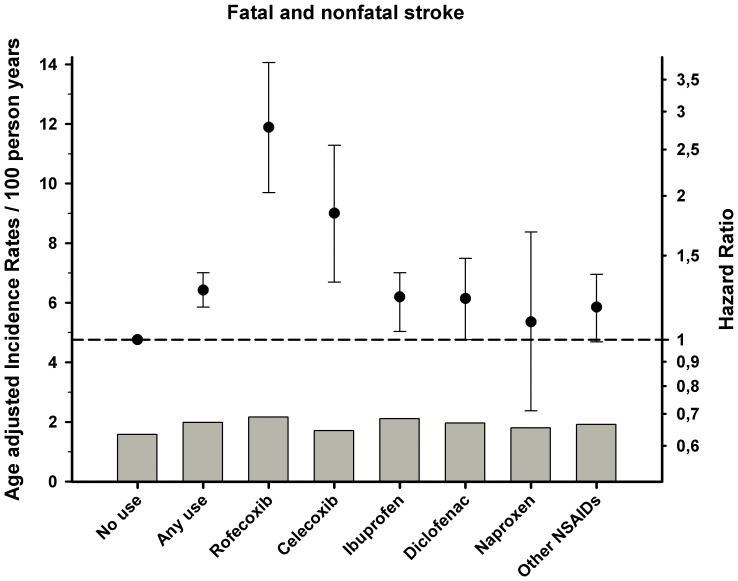
Cox proportional-hazard analysis and age adjusted incidence rates of fatal and non fatal stroke associated with use of NSAID treatment in patients with prior myocardial infarction (MI). The Cox proportional hazard model adjusted for, age, sex, year of MI, concomitant medical treatment, socioeconomic status, and comorbidity. Reference group: no use of COX-2 inhibitors or NSAIDs. Error bars indicate 95% CIs.

Incidence rates and results from the Cox proportional hazard analysis are shown in Table 3 and Figures 1, 2, 3. Overall use of NSAID was associated with increased risk of all three endpoints, but especially cardiovascular death and a composite of coronary death and non fatal MI (hazard ratio HR 1.42 95% confidence interval [CI] 1.36–1.49 and HR 1.37[1.32–1.43]), respectively).The selective COX-2 inhibitor rofecoxib was associated with an increased risk of coronary death (HR 1.65 [1.44–1.90]) and cardiovascular death (HR 1.66[1.44–1.91]), even in low doses. For the other selective COX-2 inhibitor celecoxib, the results showed a lower, yet still increased risk of cardiovascular death, coronary death, and stroke relative to rofecoxib. Use of diclofenac was associated with the highest risk of cardiovascular death and coronary death with a dose-dependent relationship relative to no NSAID use (HR 1.96 [1.79–2.15] and HR1.66 [1.51–1.81]). Use of ibuprofen showed a dose dependent association with risk of cardiovascular death and coronary death with decreased risk of coronary death in low doses and a trend for increased risk in high doses. A similar relationship was seen for ibuprofen and strokes (HR 1.23[1.10–1.38]) and diclofenac (HR 1.21[1.00–1.48]). Finally, use of naproxen was associated with the lowest risk of all the examined end points, albeit with dose-dependent increased risk in the Cox models.

**Table 3:** Crude Incidence rates for Specific Causes Associated with Exposure to NSAIDs Stratified According to Daily Dosage.

		Study population, n = 97,698 (43,049 deaths)										
	Cardiovascular Death				Coronary Death or Nonfatal MI (95% CI)				Fatal or Nonfatal Stroke (95% CI)			
Drug	No. Of Evens†	Time at risk (100 py)	Deaths IR (95% CI)	Number needed to harm NNH	No. of Evens†	Time at risk (100 py)	Events IR (95% CI)	Number needed to harm NNH	No. of Events†	Time at risk (100 py)	Events IR (95% CI)	N Number needed to harm NNH
All NSAIDs												
No use‡	21069	4300	4.96(4.89–5.02)		24093	3800	6.30 (6.22–6.38)		6756	4300	1.59(1.55–1.62)	
Any use	2252	318.79	7.06(6.78–7.36)	48 (42–55)	2420	287.56	8.42 (8.09–8.76)	47(41–56)	625	318.78	1.96 (1.81–2.12)	270(190–469)
Rofecoxib												
Any use	199	14.64	13.59(11.83–15.62)	12 ( 10–15)	204	12.97	15.73 (13.71–18.05)	11(9–13)	40	14.64	2.73 (2.00–3.72)	88( 51–329)
≤25 mg	163	13.28	12.27(10.53–14.31)	14(11–18)	172	11.78	14.60 (12.57–16.96)	12(10–16)	32	13.28	2.41 (1.70–3.41)	NA
≥25 mg	36	1.36	26.45 (19.08–36.67)	5(3–7)	32	1.19	26.95(19.01–38.10)	5(3–8)	8	1.36	5.88(2.94–11.75)	26(12–298)
Celecoxib												
Any use	189	16.87	11.21 (9.72–12.92)	16(13–21)	178	15.03	11.84 (10.22–13.72)	18(14–26)	36	16.87	2.13 (1.54–2.96)	NA
≤200 mg	113	12.57	8.99 (7.47–10.81)	25(18–41)	118	11.21	10.52(8.79–12.61)	24(17–41)	20	12.57	1.59 (1.03–2.47)	NA
≥200 mg	76	4.29	17.70(14.14–22.17)	8(6–11)	60	3.82	15.71 (12.20–20.24)	11(8–17)	16	4.29	3.73 (2.28–6.08)	47(25–289)
Ibuprofen												
Any use	906	151.32	5.99(5.61–6.39)	97 (71–155)	1009	136.12	7.41 (6.97–7.88)	90(64–151)	295	151.32	1.95 (1.74–2.19)	278 (171–732)
≤1200 mg	630	132.14	4.77 (4.41–5.16)	NA	772	118.91	6.49 (6.05–6.97)	NA	235	132.14	1.78 (1.57–2.02)	526(239–2595)
≥1200 mg	276	19.18	14.39 (12.79–16.19)	11(10–12)	237	17.21	13.77 (12.13–15.64)	14(11–17)	60	19.18	3.13 (2.4–4.03)	65843–165)
Diclofenac												
Any use	476	58.86	8.09(7.39–8.85)	32(26–41)	479	53.17	9.01 (8.24–9.85)	37(29–52)	104	58.86	1.77(1.46–2.14)	556(193–629)
≤100 mg	290	51.64	5.62(5.00–6.30)	152(77–3509)	330	46.71	7.07 (6.34–7.87)	130(66–3240)	66	51.64	1.28 (1.00–1.63)	NA
≥100 mg	186	7.22	25.77 (22.32–29.76)	5(4–6)	149	6.46	23.08 (19.65–27.09)	6(5–7)	38	7.22	5.27(3.83–7.24)	27 (19–49)
Naproxen												
Any use	76	12.26	6.20 (4.95–7.76)	NA	96	11.32	8.48 (6.94–10.36)	46(26–180)	21	12.26	1.71 (1.12–2.63)	833(118–165)
≤500 mg	43	9.31	4.62 (3.43–6.23)	NA	63	8.71	7.24 (5.65–9.26)	NA	9	9.31	0.97 (0.50–1.86)	NA
≥500 mg	33	2.95	11.19 (7.95–15.74)	16(10–38)	33	2.62	12.62 (8.97–17.75)	16(10–44)	12	2.95	4.07 (2.31–7.16)	40(21–445)
Other NSAIDs												
Any use	519	73.83	7.03(6.45–7.66)	48(38–67)	550	66.99	8.21(7.56–8.93)	52(39–80)	152	73.83	2.06(1.76–2.41)	213(126–695)

† No. of events while having drug available for treatment.

‡ Reference group.

### Sensitivity analyses

Almost half of the individuals who died during NSAID treatment died of non cardiovascular causes. We therefore performed sensitivity analyses, which showed a lower incidence rate of dying of non-cardiovascular causes while taking NSAIDs (incidence rate [IR] 2.17 95% CI2.01–2.35]) compared to the IR of cardiovascular death (IR 7.06 [6.78–7.36]).Overall, incidence of non cardiovascular conditions such as infections were low after NSAID treatment was initiated(data not shown).There was an increased risk of death from malignancy with NSAIDs compared to nonusers in the COX models, but here the IR was two times lower than for cardiovascular death(data not shown).

The effect of unmeasured confounders cannot be excluded. Our calculations showed that if an unmeasured confounder or a combination of confounders was present in 20% of the cohort treated with NSAIDs, the confounder would have to elevate the risk by a factor 3.4 to 3.8 to explain the increased risk observed in our study.

We performed a sensitivity analyse examined if the increased risk with overall NSAID and with the individual NSAID persisted after stopping treatment. We divided the periods of not taking NSAID into time intervals of 14, 30, 90 days and used the Cox-proportional hazard models. For overall NSAID use the increased risk returned to baseline shortly after treatment was discontinued. For the individual NSAIDs the same trend was seen with diclofenac and ibuprofen, but for the selective Cox-2 inhibitor rofecoxib the risk was persistently increased after stopping treatment (data not shown). Aspirin is available over the counter, which may explain why the fraction of patients who fill prescriptions for aspirin is relatively low. Another consequence of this is that we do not have information on whether using NSAIDs may lead to prematurely discontinuing aspirin. However to analyse whether NSAIDs user had more bleeding we made a sensitivity analyse, where we censored patients at bleeding. This did not change the results.

## Discussion

This study examined cause-specific cardiovascular mortality and morbidity associated with NSAID treatment in a population of patients surviving to 30 days after their first-time MI. Utilization of all NSAIDs was associated with an increased risk of cardiovascular mortality and morbidity, yet only in high doses for ibuprofen and naproxen.The novel finding of this study is that we found different cause-specific cardiovascular risks associated with NSAID use among patients with prior MI.

In observational studies as ours one can not exclude the possibility that the high risk associated with use of NSAIDs was due to confounding by indication, i.e., patients taking NSAIDs may generally be sicker than those not treated with NSAIDs. However in the present study we found that deaths associated with NSAIDs were mainly due to cardiovascular cause. We found a clear relationship between the degree of COX-2 inhibition, and a dose dependent increase in risk, again indicating that the effect was associated with the drugs rather than with their therapeutic indications. This result further strengthens the known association between use of NSAID and cardiovascular risk. Rofecoxib, celecoxib, and diclofenac are all characterized by a high degree of COX-2 selectivity and our findings could therefore be suggestive of a detrimental COX-2 inhibitor class effect on cardiovascular outcomes [14]. Various mechanisms, for instance hypertension, heart failure, renal failure and increased tendency for thrombosis have been proposed to explain an increased risk with COX-2 selective NSAIDs, with emphasis on their considerable inhibition of prostacyclin synthesis but failure to inhibit COX-1-mediated generation of thromboxane A2 in platelets (which are devoid of COX-2) [15], [16], [17], [18], [19], [20].

Several studies have confirmed increased cardiovascular risk associated with rofecoxib and diclofenac, and general treatment recommendations include caution with NSAIDs and avoidance of selective COX2-inhibtors if possible [15], [21], [22] Indeed, patients with established cardiovascular disease or patients at increased cardiovascular risk seem to be more vulnerable to the cardiovascular toxicity of NSAIDs. An interaction between previously cardiovascular event and NSAID use is found, however the effect size of this still remains unknown. In the present study we are looking at patients with first-time MI, and therefore we aren't able to analyze this interaction. However we have previously demonstrated that NSAID use among patients with first-time MI was associated with persistently increased risk of all-cause mortality and of a composite of coronary death or nonfatal recurrent MI, respectively, for at least 5 years thereafter. These results support previous findings that NSAIDs have no apparent safe treatment window among patients with MI. However even healthy people have increased risk of cardiovascular diseases taking NSAIDs in particular diclofenac. Fosbøl et al. have previously analyzed the cause-specified cardiovascular risk associated with NSAID in healthy individuals [23]. In brief, they found that most NSAIDs were associated with increased cardiovascular risk and in particular, the use of the diclofenac and rofecoxib was associated with increased risk of cardiovascular mortality and morbidity. The present study compliments these findings in a high-risk population of post-MI patients where patients were generally more elderly, more frequently treated with NSAIDs, and with a high risk of subsequent cardiovascular events. The potential interaction between previously cardiovascular disease and NSAID needs further investigation, especially clarifying the effect size of this. Because treatment with NSAIDs is so prevalent in the general population, it is of great importance that the safest treatment alternative among the NSAID group is found when NSAID treatment cannot be avoided, and therefore further studies, specially randomized trials, are required. Of most concern may be the fact, that the traditional NSAID diclofenac, which is one of the most frequently used NSAIDs (available OCT in many countries), and where the considerable COX-2 selectivity may not be generally recognized, was associated with one of the highest risks of cardiovascular morbidity and mortality including fatal or nonfatal stroke and coronary death.

In our study, the NSAID with the least cause-specific cardiovascular risk was naproxen and this result is in accordance with previous studies [20], [24]. The cardiovascular safety of naproxen has been questioned, as a recent randomized study, designed to investigate the effect of the agent on Alzheimer dementia, was terminated due to a possible excess of adverse cardiovascular events with naproxen [25]. Ibuprofen is widely sold OCT, which may lead to the assumption that this is a particularly safe NSAID. However in a recent meta-analysis by Trelle et al. the highest risk of stroke was associated with use of ibuprofen [22]. The same trend was seen by Fosbøl et al, where use of ibuprofen treatment was associated with a trend for increased risk of fatal and nonfatal stroke [23]. We found a non-significant trend for increased cardiovascular risk, with a dose-dependent relationship, associated with ibuprofen therapy. Thus, considering the current results and the accumulated evidence at this point, naproxen may be a safer alternative to ibuprofen in patients requiring NSAID treatment.

### Strengths and limitations

This study's main strength was its completeness and size of data. This complete registration, including all citizens independent of race, socioeconomic status, age or participation in health insurance, also those outside the labour market, insured a minimum of selection bias. The Danish National Register as well as the national prescription register is known to be accurate [6], [26]. The data on mode of death were obtained from the Causes of Death Register, which is based on information from the death certificate filled out by the doctors declaring the individual's death or the individual's general practitioners with knowledge on the patient's comorbidities, if unknown by the doctors declaring the individual's death.

Complete registration was ensured as all Danish pharmacies are required to register all dispensed drug prescriptions [26]. In Denmark, the only NSAID that was available as OTC drug during the study period, was ibuprofen (sine 2001), but only in low dosage (200 mg) and in limited quantity (100tablets). Furthermore in Denmark there is partial patient copayment of drug expenses and patients needing higher doses or long-term treatment would have a financial incentive to obtain a prescription from their physician to receive reimbursement. It is therefore unlikely that OTC use of NSAIDs had a major impact on this study.

The main limitation of our study was the observational nature of the study. We do not have any information about the precise indication for initiation of NSAID treatment and the calculations of dose and treatment duration represent approximations. The Cox models were used provide control of available confounders, but the control for confounding by indication may not have been complete. For this reason, we performed supplementary analyses investigating the risk of infectious disease, malignancies and deaths from other causes. We found low incidence rates of all these death causes associated with NSAID use. The observed dose-response effect, the different relative effects of different NSAIDs used for similar indications, and the clear relation between the degree of COX-2 inhibition as reported in the literature and risk of adverse cardiovascular outcomes observed here all support the importance of NSAIDs and not of the potential confounders. Furthermore, important cardiovascular risk factors such as lipid levels, smoking status, blood pressure, left ventricular ejection fraction, or obesity were lacking. Therefore we made a Schneeweiss analysis, which showed that if an unmeasured confounder or a combination of confounders was present in 20% of the cohort treated with NSAIDs, the confounder would have to elevate the risk by a factor 3.4 to 3.8 to explain the increased risk observed in our study. The existence of such a confounder or combination of confounders is highly unlikely, but not entirely impossible as we had no information on other important risk factors such as smoking, lipid levels, or body mass index.

Another limitation is the classification of death. The Causes of Death Register is based on information obtained from the death certificate filled out by the doctor declaring the individual's death. All deaths are registered in the Causes of Death Register and data are by legislation complete. In prior studies validation of coronary and cardiovascular events demonstrated acceptable levels of sensitivity, with a tendency to overestimate cardiovascular deaths, although this overestimation would occur in all risk groups in our study [27], [28]. The specific diagnosis of MI (combined validity of fatal and nonfatal MI) has proved to be valid, with a sensitivity of 91% and a positive predictive value of 93%. Through records on all hospital admissions to Danish hospitals the nonfatal events are obtained, whereas the fatal events are recorded in the Causes of Death Register based on information gathered from the death certificate. Nonfatal events, which did not result in hospital admission would not be recorded and this could in theory introduce bias; however, this is unlikely to influence the results, given the nature of the nonfatal events studied (i.e., MI and stroke). The stroke diagnoses (both fatal and nonfatal) used in this study has also been validated with a good result, and it was found that the diagnoses had positive predictive values of 74% to 97% [12], [13].

Aspirin is available over the counter, which explains why the fraction of patients who fill prescriptions for aspirin is relatively low. Another consequence of this is that we do not have information on whether using NSAIDs may lead to prematurely discontinuing aspirin. We assume that most patients who did not fill a prescription for aspirin were treated with over-the-counter aspirin, since medication adherence has been documented to be high among patients in Denmark after MI [29]. No study design can exclude the possibility that individuals do not take all prescribed medications and prescription data must therefore be viewed in the light of the possibility of non-adherence. However, non-adherence would influence the results towards the null and hence dilute the observed association between NSAID exposure and adverse cardiovascular outcomes. Another limitation is the effect of information bias. The patients don't necessarily take their medications consecutively leading to the fact that the prescription may run longer and the patients therefore are exposed later than the database might indicate. There would be no measurable consequences for the rest of the population since data from individuals taking therapy without being noted to be on a prescription would be diluted in the data from the much larger population not on therapy. However to control for this phenomenon, we examined if the increased risk with overall NSAID and with the individual NSAID persisted after stopping treatment. We divided the periods of not taking NSAID into time intervals of 14, 30, 90 days and used the Cox-proportional hazard models. For overall NSAID use the increased risk returned to baseline shortly after treatment was discontinued. For the individual NSAIDs the same trend was seen with diclofenac and ibuprofen, but for the selective Cox-2 inhibitor rofecoxib the risk was persistently increased after stopping treatment. This latter observation appears to strengthen our study conclusions even more as the same result with rofecoxib was very recently found in an analysis of data from a previous clinical trial [30].

## Conclusions and Clinical Implications

This nationwide study of a post-MI cohort demonstrated that individual NSAIDs exert different cause-specific cardiovascular risks. In particular rofecoxib and diclofenac were associated with high risk of cardiovascular specific morbidity and mortality. Our results also showed a dose-response relationship between NSAIDs dosages and adverse cardiovascular outcomes. Further studies and preferably randomized trials are warranted to establish the cardiovascular risk associated with individual NSAIDs in subgroups of patients with cardiovascular disease and other populations. Although our study was based on observational data, it provides additional support for increased cardiovascular risk associated with NSAID treatment in post-MI patients and advocates caution with any use of NSAIDs in patients with prior MI.
